# Adipose-derived stem cells (MYSTEM® EVO Technology) as a treatment for complex transsphincteric anal fistula

**DOI:** 10.1007/s10151-018-1785-2

**Published:** 2018-05-02

**Authors:** P. Lobascio, G. Balducci, M. Minafra, R. Laforgia, S. Fedele, M. Conticchio, N. Palasciano

**Affiliations:** General Surgery Unit “V. Bonomo”, Department of Emergency and Transplantation of Organs, University of Bari, P.zza G. Cesare 11, 70124 Bari, Italy

## Introduction

High fistula-in-ano is a challenge for coloproctologists. Several surgical procedures to treat these fistulas have been developed in recent decades. Loose (or cutting) seton, fistulotomy, ligation of the intersphincteric fistula tract (LIFT) or fistulectomy with endorectal flap are considered sphincter-preserving techniques, but patients complain about discomfort and the prolonged healing period. Application of fibrin glue is considered a valid technique in selected patients. A new frontier in the treatment of perianal fistulas is the application of adipose tissue-derived stem cells (ADSCs). There are many sources of adult stem cells including the bone marrow, but the discovery of ADSCs paved the way for their large-scale use [1]. They can be easily obtained from adipose tissue with minimally invasive techniques which provide a high percentage of stem cells and very low risk of stem cell damage [2]. ADSCs are multipotent and can differentiate into various cell types, and are characterized by immunosuppressive properties and low immunogenicity. Multipotent mesenchymal stem cells [3] and ADSCs [4–6] have been used for autologous transplantation in the treatment of fistulas in patients with Crohn’s disease over the last few years with good results. This is a sphincter-preserving technique and avoids the risk of fecal incontinence associated with conventional management. We describe our experience with MYSTEM® EVO Technology (MySTEM LLC, Wilmington, DE, USA) in an autologous transplantation of ADSC for closure of a complex transsphincteric fistula. The common procedures for adipose-derived stem cell isolation are mainly based on tissue fractionation and enzymatic digestion, requiring many hours, making it unsuitable for direct surgical applications. Recent studies demonstrated the feasibility of isolating adipose stromal cells without the need for enzymatic digestion. These studies reported the processing of the fluid portion of liposuctioned adipose tissue (lipoaspirate fluid), which contains a significant amount of progenitor cells endowed with plastic and trophic features.

In our case, we introduce a brand new closed device, MYSTEM® EVO Technology, which allows nonenzymatic tissue separation and rapid isolation of lipoaspirate fluid from human liposuctioned adipose tissue.

## Clinical case

**Fig. 1:**
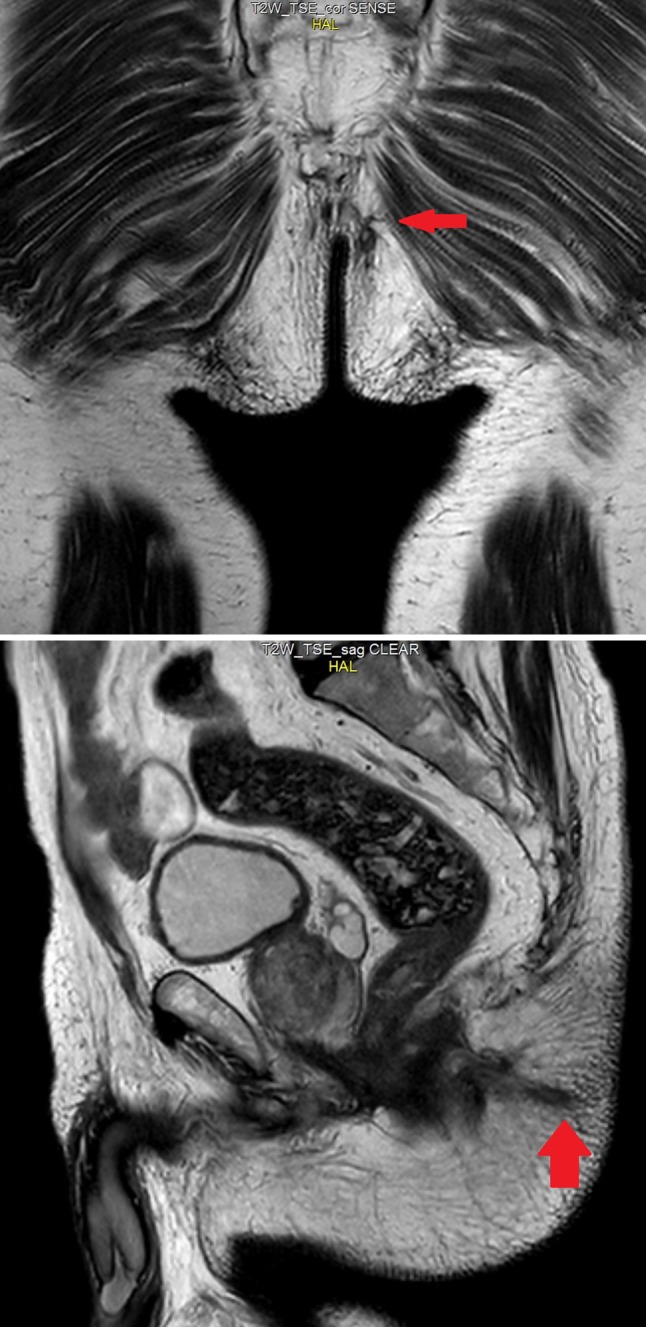
Pelvic magnetic resonance imaging with evidence of transphinteric fistula (red arrow) of the high-mid anal canal without any internal opening and another fistula extending as far as the left internal obturator muscle

**Fig. 2:**
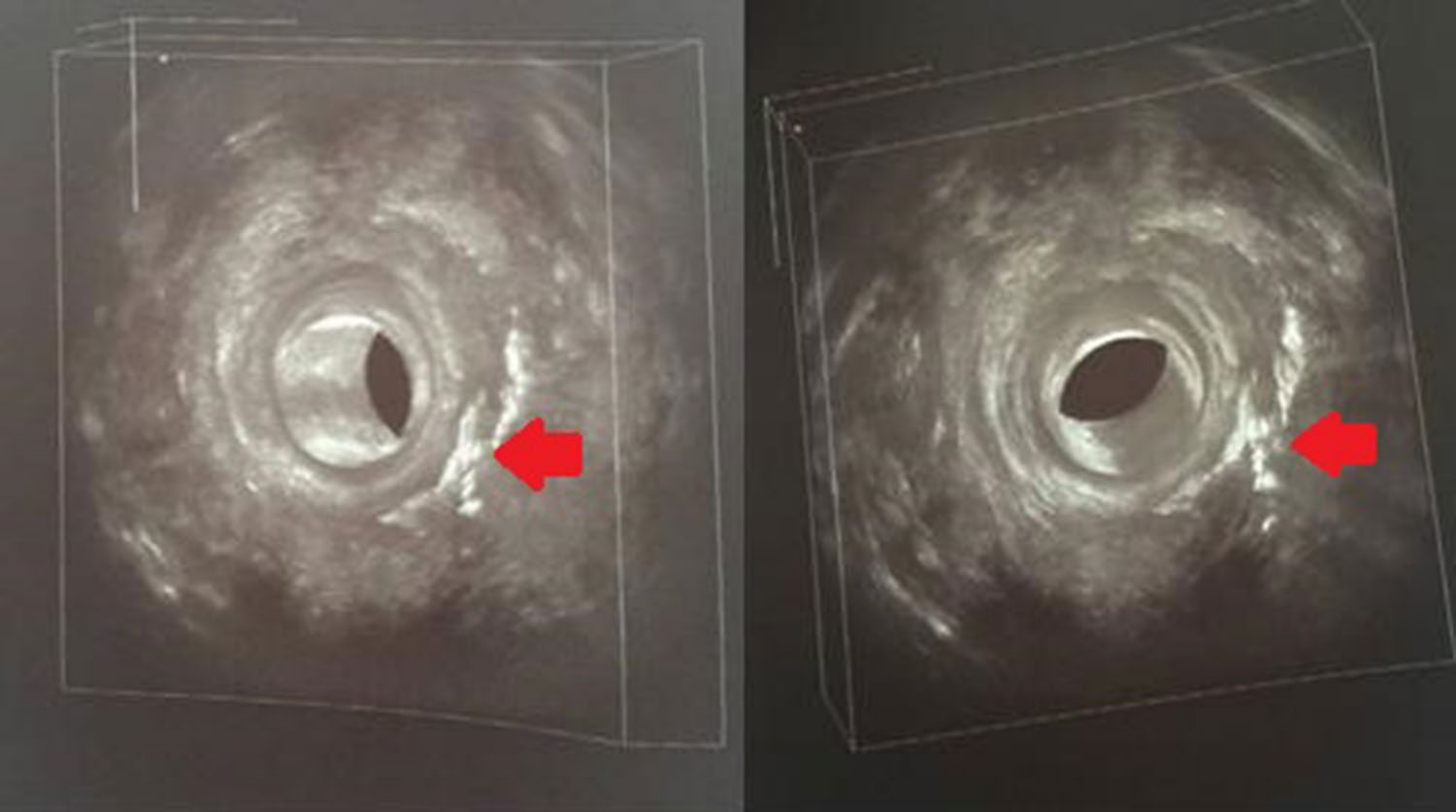
Preoperative transanal ultrasound showing the fistula (red arrow)

We present the case of a 77-year-old male presenting with a left posterior lateral perianal abscess associated with a 6 cm long fistula tract and a posterior external opening without an internal opening. Previously, the patient had undergone abscess draining and fistulotomy. Transanal ultrasound and magnetic resonance imaging confirmed a transsphinteric fistula in the high-mid anal canal without any internal opening, and another fistula extending as far as the left internal obturator muscle (Figs. 1, 2). The patient was selected for ADSCs treatment and preoperatively prepared with daily lavage using hydrogen peroxide and sodium chloride solution. The surgical procedure was performed when the fistula had stopped discharging pus. MYSTEM® EVO Technology (MySTEM LLC, Wilmington, DE,USA) was used to obtain stem cells.

### Abdominal step

**Fig. 3:**
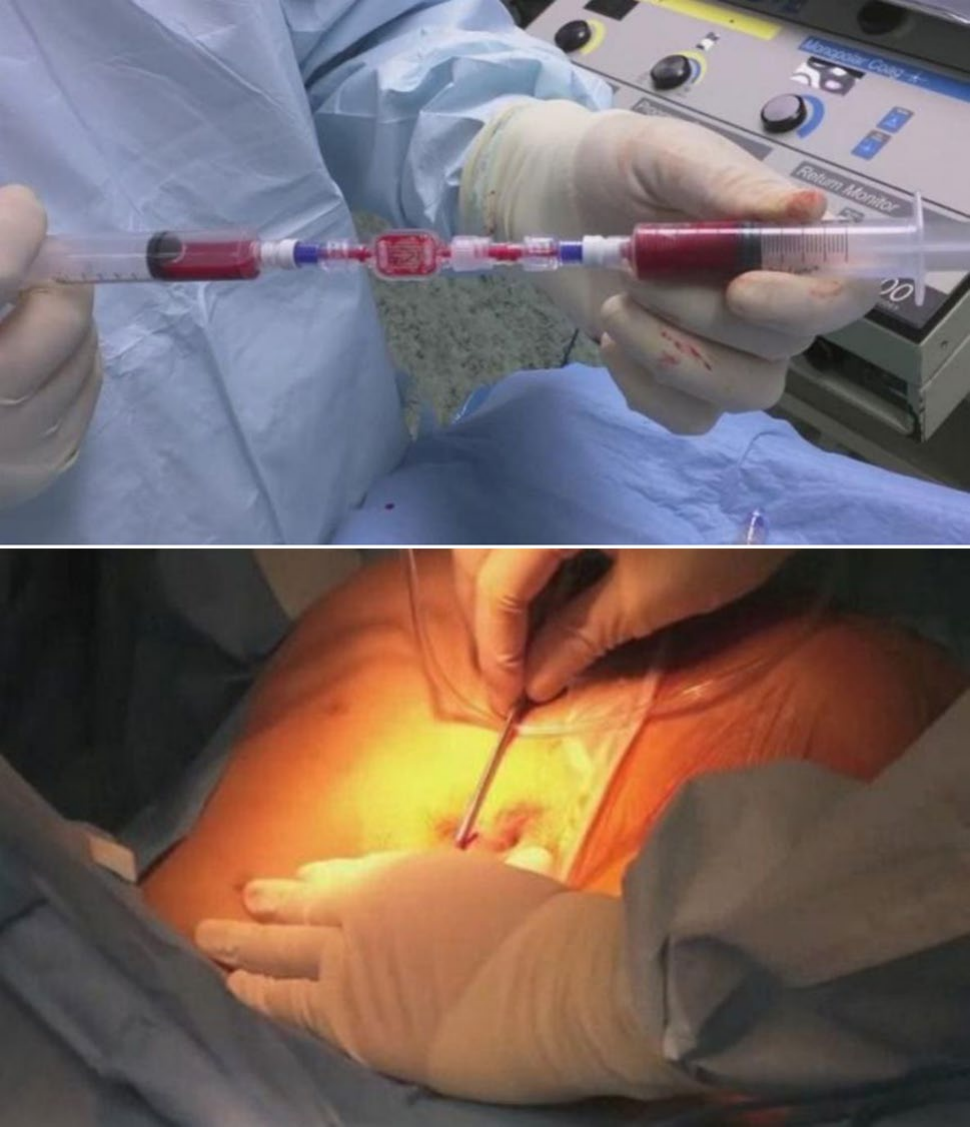
MYSTEM® EVO Technology (MytSTEM LLC, Wilmington, DE, USA) procedure used to obtain stem cells

Paraumbilical incision (2 cm) and liposuction of abdominal tissue (8 ml) were performed to obtain adipose cells. This harvested tissue underwent the MyStem® CVF Isolation Process: fractioning, separation from lipoaspirate fluid and then concentration to achieve intact adipose tissue lobules, with morphologically preserved cellular membranes (Fig. 3). This procedure was repeated four times.

### Perineal step

The external opening was identified (the internal opening was not found, in concordance with the imaging). Meticulous brushing and curettage were completed and the fistula was explored with a fistula probe. Then 10 ml of ADSCs obtained with MyStem was injected from outside to inside, directed toward the hypothetical internal opening. Only one injection (10 ml of ADSCs) with a 21 G hypodermic needle was performed inside the fistula using the external opening. The external opening was closed with a 3–0 Vicryl stitch.

Recovery was uneventful and the patient was discharged on postoperative day (POD) 2. The patient was kept overnight as it was the first procedure of this kind that we had performed. Normally a patient can be discharged in POD 1 or the same day if there is a Day Hospital service.

**Fig. 4:**
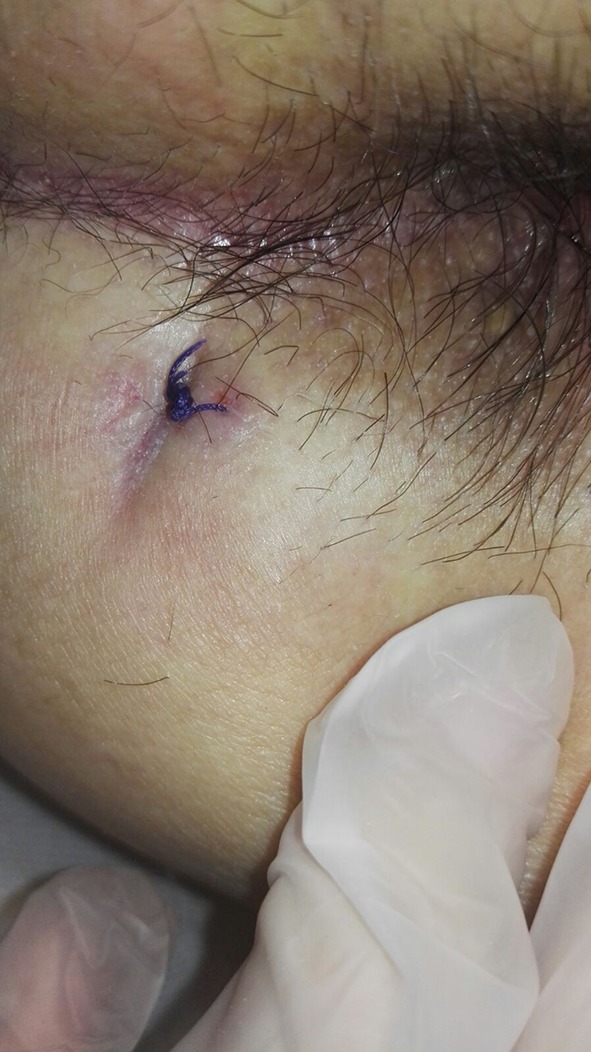
Appearance of surgical wound at follow-up on postoperative day 7

**Fig. 5:**
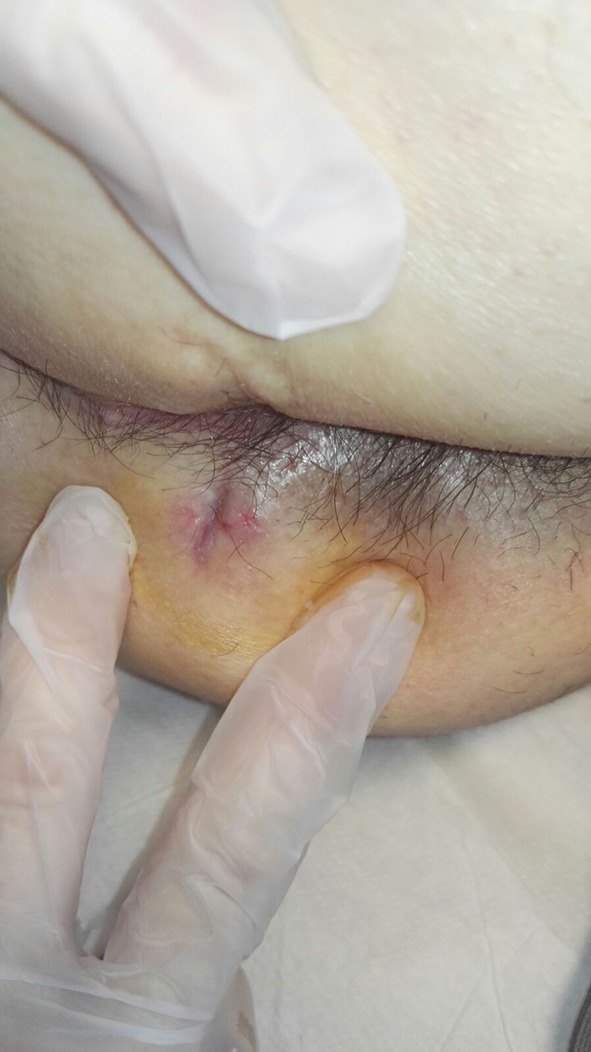
Appearance of surgical wound at follow-up on postoperative day 10

**Fig. 6:**
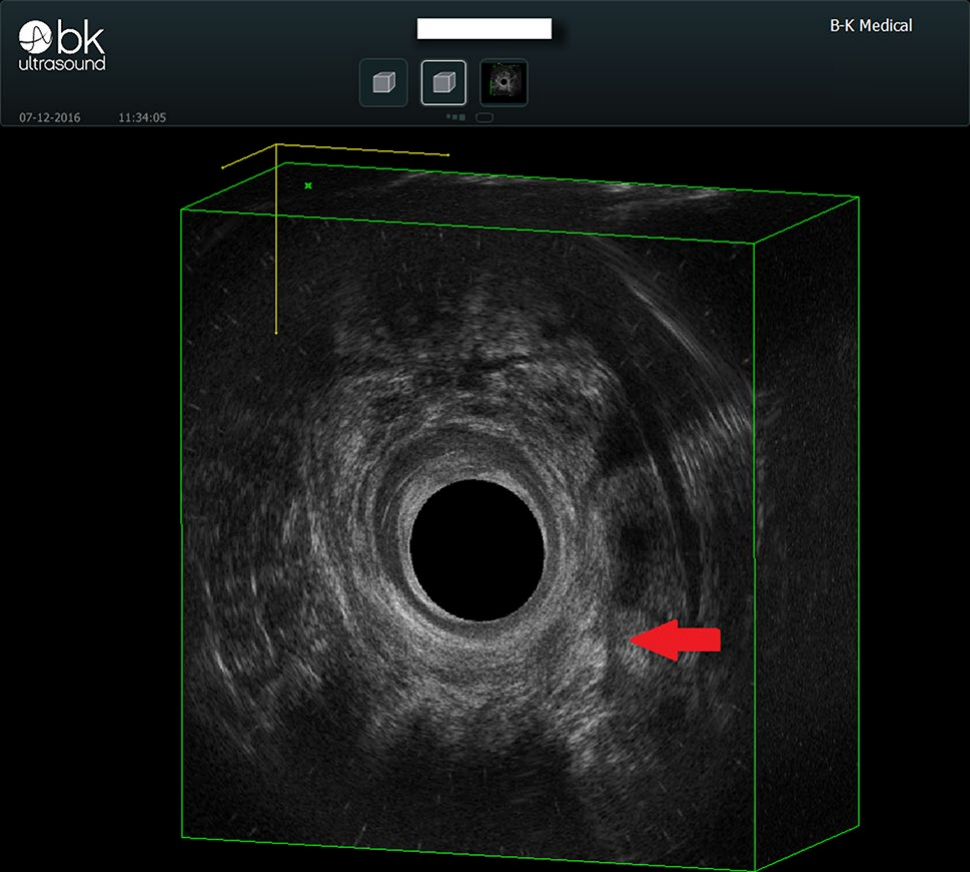
Transanal ultrasound follow-up showing complete healing of the fistula (red arrow) at 30 days after surgery

**Fig. 7:**
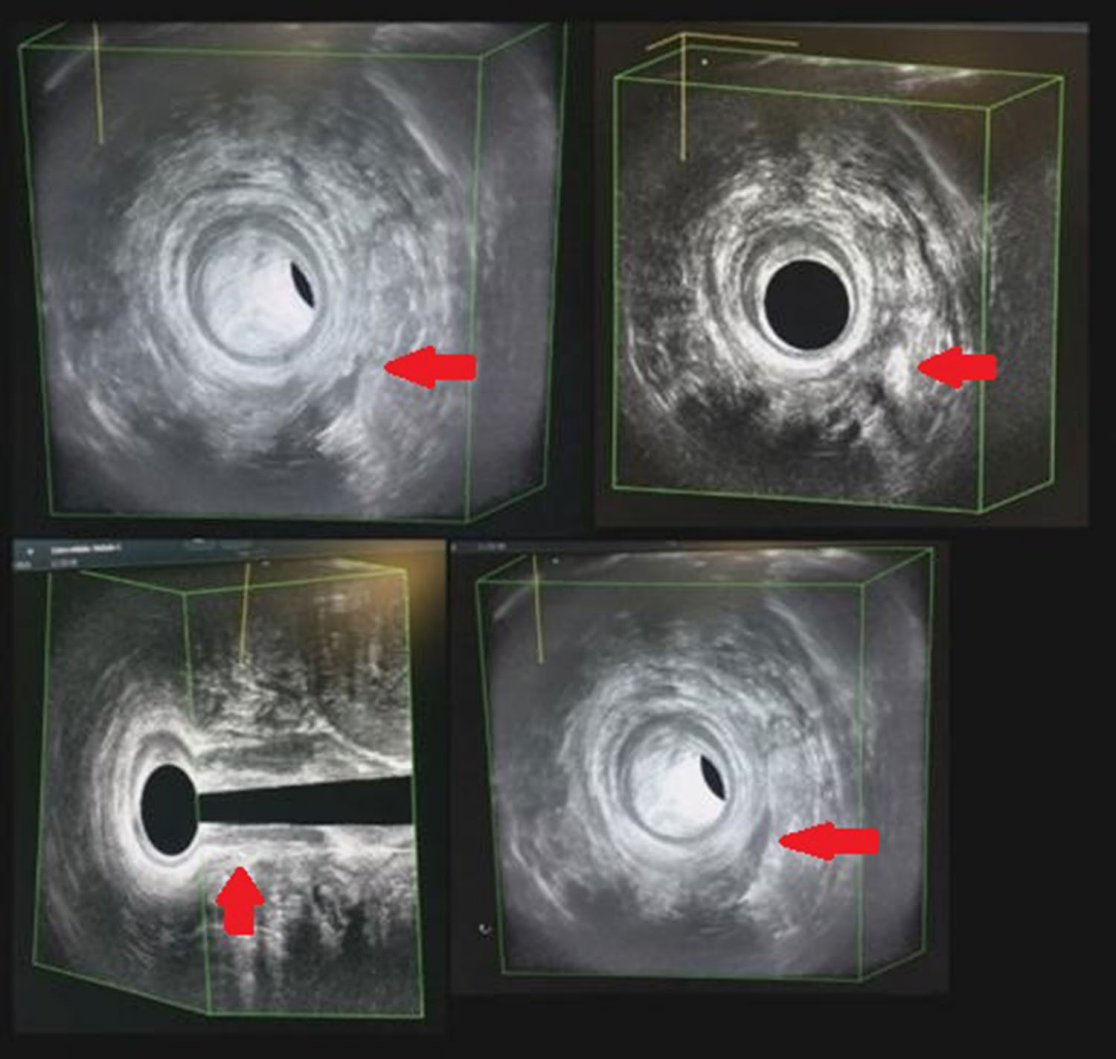
Transanal ultrasound follow-up showing complete healing of the fistula (red arrow) at 6 months after surgery

The patient was followed-up at 7, 10, 30 and 180 days after the procedure, with anoscopy and transanal ultrasound demonstrating complete healing of the fistula (Figs. 4, 5, 6, 7). At one year of clinical follow-up the fistula remains healed.

## Discussion

Adipose tissue is an excellent source of adult stem cells, because of plasticity features which have important potential for several future applications. The most common ADSC protocol is based on liposuction, which separates lipoaspirate into fatty and fluid portions, both of which contain adipose-derived stromal cells In vivo studies have demonstrated that stem cells from the fluid portion have a more promising role in tissue reconstruction than those derived from the fatty portion [1, 2]. Due to the anti-inflammatory and immunomodulatory capacity of stem cells, stem cell-based therapies may well have a revolutionary impact on the management of Crohn’s fistulas [4, 7]. In addition to the treatment of refractory Crohn’s fistulas, stem cell-based therapy was tested in a complex perianal fistula in a randomized study which demonstrated that the application of expanded ADSCs in combination with fibrin glue is an effective and safe treatment and appears to achieve higher rates of healing than fibrin glue alone [5].

To our knowledge, this is the first case of cryptogenic anal fistula treated with injection of stem cells obtained by MYSTEM® EVO Technology.

## Conclusions

Our experience suggests that ADSCs are a promising new sphincter preserving treatment option for high or complex transsphincteric anal fistulas, and that MYSYSTEM® EVO Technology is potentially useful for this application.
